# Effect of Silica Nanoparticles on Polymer Adsorption Reduction on Marcellus Shale

**DOI:** 10.1021/acsomega.1c03653

**Published:** 2021-10-29

**Authors:** Sameer Al-Hajri, Berihun M. Negash, Md Motiur Rahman, Mohammed Haroun, Tareq M. Al-Shami

**Affiliations:** † Petroleum Engineering Department, Khalifa University, Abu Dhabi 127788, United Arab Emirates; ‡ Shale Gas Research Group, Institute of Hydrocarbon Recovery, Universiti Teknologi PETRONAS, Seri Iskandar, Perak 32610, Malaysia

## Abstract

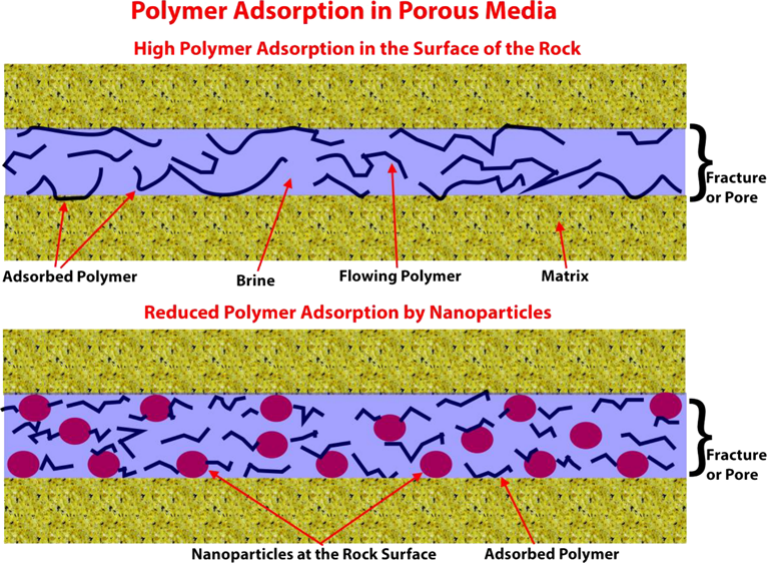

Polymers play a major role in developing rheology of fracturing fluids for multistage hydraulic fracturing horizontal wells in unconventional reservoirs. Reducing the amount of polymer adsorbed in the shale formation is essential to maintain the polymer efficiency. In this study, the ability of silica nanoparticles to minimize polymer adsorption in Marcellus shale formation at reservoir temperature was investigated. Partially hydrolyzed polyacrylamide polymers of varying molecular weights (1–12 MD), salinities (2500–50,000 ppm), polymer concentrations (100–2000 ppm), and silica nanoparticle concentrations (0.01–0.1 w/w) were used in the static adsorption experiments. Adsorption of the polymer in the Marcellus shale samples was contrasted with and without the silica nanoparticles at a Marcellus formation reservoir temperature of 65 °C, showing a significant polymer adsorption reduction of up to 50%. The adsorption and adsorption reduction were more sensitive to the variation of the polymer concentration than to the variation of the salinity within the tested conditions. The highest adsorptions were reported at the higher molecular weight of 10–12 MD. In addition, silica nanoparticles significantly improved polymer rheology at elevated temperatures. The results indicate that nanoparticles can play a significant role in reducing polymer adsorption in the fracturing fluid and improve its rheological properties and its efficiency, which will reduce the number of issues caused by the polymers in the fracturing fluid and making it more cost effective.

## 1 Introduction

Due to the increase in the demand for energy, unconventional hydrocarbon reservoirs will play a significant role in satisfying global demands for hydrocarbon in general and gas in particular in the future.^1,2^ The most important resource for unconventional hydrocarbons is the shale reservoirs. However, developing shale reservoirs is more expensive than conventional reservoirs. Advanced and sophisticated technologies are required to produce hydrocarbons at an economical rate.

Conventional resources include migration and accumulation in sandstone or carbonate porous formations. The unconventional hydrocarbon source rock, however, is the same as the reservoir in which the organic matter was buried and cooked at high temperature and high pressure over a very long time.^3^ The challenge in evaluating unconventional reservoirs is that for all petrophysical calculations of the reservoir properties, the kerogen or organic matter properties should be included in all of the calculations.^4^ This organic matter goes through stages of development from organic matter to finally oil or gas. This in fact also helps in forming extra porosity in the source rock where hydrocarbon is stored. Results from low-temperature nitrogen adsorption/desorption and mercury intrusion porosimetry tests normally yield nanoscale to microscale porosity, which are combined to give a better estimation of the porosity. The only drawback of these two methods is that they are only able to detect the interconnected pores. In contrast, nuclear magnetic resonance is able to provide a pore size distribution of the immersed sample with liquid and show the pores filled with liquid, regardless of the connectivity of the pores.^5^ Furthermore, another significant feature of unconventional reservoirs is the shale brittleness, which is a mechanical behavior that helps to find sweet spots for hydraulic fracturing operations.^6^

One of the essential techniques for developing a shale reservoir is hydraulic fracturing.^7^ The process involves injecting a fracturing fluid into the shale formation at high pressures to create fractures/cracks in the deep rock formations. The fracturing fluid primarily contains water, sand, thickening agents such as polymers, and other additives.

Polymers play a significant role in the success of a fracturing process.^8,9^ Polymers have many functions; they serve as viscosifiers of the fracturing fluid to reduce friction with the tubing and provide an adequate suspension of the proppant.^10^ They also serve as a coating agent for the proppant sands to improve their mechanical properties and prevent the proppant flow back.^11^ Similarly, the width of the induced fracture in the shale formation is controlled by the rheological properties of the fracturing fluid (polymer rheology).^12^ However, the polymer can be adsorbed in the shale formation, which may reduce the concentration of the polymer in the fracturing fluid. As a result,the polymer efficiency will be reduced.^13^

Polymer adsorption in shale formations is unfavorable because the carrying capacity of the fracturing fluid will be decreased.^14^ Polymer adsorption depends on various factors, such as the polymer’s concentration and molecular weight, surface area, salinity, and temperature.^15−17^ Moreover, polymer adsorption will be significantly affected by the shale rock mineralogy during hydraulic fracturing. However, the shale reservoir is highly heterogeneous, and mineralogy is variable. Thus, an adequate estimate of polymer adsorption is difficult.^13^ Therefore, it is of great advantage to reduce polymer adsorption. One of the effective methods to reduce chemical additives’ adsorption used in enhanced oil recovery in sandstone and carbonates is the use of nanoparticles.^18,19^ The nanoparticles are also used to reduce surfactant adsorption in the rock surfaces.^19^ This assumes that the nanoparticles remaining in the porous media will provide a kind of a shield on the sand surface, and the molecules of the surfactant could collide on the surface of these nanoparticles, thus decreasing the adsorption of the surfactant.

Nanoparticles or their manipulations are very small materials, at the range of about 1–100 nanometers in diameter.^20−22^ Recently, nanotechnology has been a significant focus in the petroleum industry because the addition of nanoparticles may change the properties of drilling fluid, strengthening sand consolidation, and improves drag reduction.^23−32^ However, the role of nanoparticles in the reduction of polymer adsorption on shale surfaces has not been reported in the literature. In this paper, an investigation on the role of silica nanoparticles in the adsorption process on Marcellus shale samples is conducted. Static adsorption tests were conducted for three partially hydrolyzed polyacrylamide polymers of low molecular weight (LMW), medium molecular weight (MMW), and high molecular weight (HMW). The study was also conducted using different nanoparticle salinities and concentrations and at a reservoir temperature of 65 °C. The temperature was reported to be a representative temperature for the Marcellus shale reservoir with depths of 4000–8500 ft.^33^ The rheology of the three polymers was also investigated with and without the nanoparticle at elevated temperatures.

## 2 Materials and Methods

### 2.1 Polymer Solution

Ultrapure water was used for all the experiments. Partially hydrolyzed polyacrylamide (HPAM) was used in this research. The polymer was supplied as a white granular powder. The polymer solution was prepared by mixing and dissolution using a magnetic stirrer. First, KCl of 50,000 ppm was added to the water in a beaker with a magnetic stirrer to make the base solution for the different concentrations. The solution was then stirred for 2 h and next diluted with ultrapure water to yeild solutions of 2500, 10,000, 30,000, and 50,000 ppm of KCl. Then, the polymer powder was introduced slowly to prevent fish-eye formation or agglomeration of the polymers. The solution was then stirred slowly for 24 h for complete dissolution. Finally, silica nanoparticles were added to the polymer solution and stirred using an ultrasonic mixer for dispersing, homogenizing, and mixing of the polymer solution and the silica nanoparticle. The silicon dioxide (SiO_2_) nanoparticles are nearly spherical in shape. The compositions and concentrations of all samples prepared are shown in Table 1.

**Table 1:** Compositions and Concentrations of the Samples Used in This Study

polymer	MW (MD)	polymer concentration (ppm)	salinity (ppm)	silica concentration (w/w)
Flopaam 3130 S	1–2	100, 500, 1000, and 2000	2500, 10,000, 30,000, and 50,000	0.01, 0.05, and 0.1
Flopaam 3330 S	5–6	100, 500, 1000, and 2000	2500, 10,000, 30,000, and 50,000	0.01, 0.05, and 0.1
Flopaam 3630 S	10–12	100,500, 1000, and 2000	2500, 10,000, 30,000, and 50,000	0.01, 0.05, and 0.1

The rheology of the three HPAM was determined using the thermal analysis and rheology instrument at shear rate ranges of 0.1–500 s^–1^, and the viscosity measurement was conducted at 25, 45, 65, 85, and 105 °C.

### 2.2 Sand

The sand was prepared by crushing and sieving samples of the Marcellus shale. Sand particles with a diameter of 80–125 microns were chosen for the experiments. The sand was then washed in a beaker with constant shaking of the beaker until the tiny floating particles (mudlike) were isolated and removed. This was done using a careful step-by-step process to remove the fine particles, since they affect the resulting polymer adsorption. The steps were repeated until all the small particles were removed, and the water appeared free of floating particles.

### 2.3 Adsorption Test

**Figure 1:**
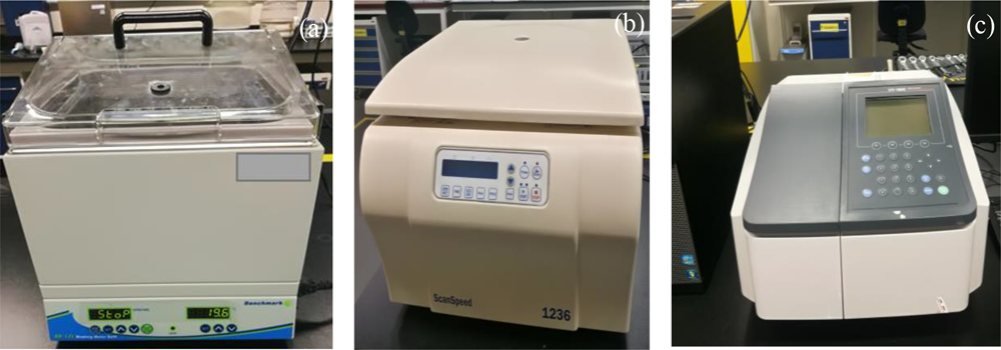
Apparatus used for the polymer adsorption test; (a) water bath; (b) centrifuge; and (c) UV–vis.

The prepared sand was then added to the polymer solution in a beaker to have a solid-to-liquid ratio of 0.1. The beaker containing the adsorbent and adsorbate was placed and agitated in a water bath for 4 h for adsorption at 65 °C. The solid and liquid elements were manually separated. However, some solid particles remained in the liquid. To remove the rest of the sand particles from the liquid, a centrifuge was used. Finally, the collected polymer solution was taken for UV–vis measurements. The UV–vis results are the absorbance results (wavelength between 1100 and 190 nm) of each sample used in the static test. The peak of each absorbance curve was then converted into concentrations using the calibration curves created from known concentrations. Figure 1 shows the water bath, centrifuge, and UV–vis instrument used in this study.

Polymer adsorption was then calculated using eq 1:^34,35^
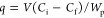
1where *q* is the adsorption concentration, μg/g; *V* is the volume of the polymer solution, mL; *C*_i_ is the initial polymer concentration, μg/mL; *C*_f_ is the polymer concentration after the static adsorption, μg/mL, and *W*_p_ is the weight of the crushed core in grams.

## 3 Results and Discussion

### 3.1 Marcellus Shale Surface Area

The adsorption in the Marcellus shale sample is proportional to its grains’ surface area because of the fact that adsorption is an interaction between polymer molecules and the rock surface. Thus, determining the surface area of the Marcellus shale sample is very important for predicting the extent of the polymer adsorption. Particle size with a diameter of 80–125 microns was used for the Brunauer–Emmett–Teller (BET) test, which is similar to the particle size used in the static adsorption test to ensure consistency of our results.

The surface area of the Marcellus sample obtained using a BET surface area analyzer at low-pressure nitrogen gas adsorption is 15.37 m^2^/g. This value is consistent with the literature reporting the surface area of the Marcellus shale ranging from 10 to 25 m^2^/g.^36^ This indicates that the sample has high potential for adsorption of the polymer. Prior to the BET analysis, the samples were placed in an oven, controlled at 150 °C under a vacuum of 10^–6^ Pa for more than 24 h to degas and remove moisture.

### 3.2 Mineralogy

**Figure 2:**
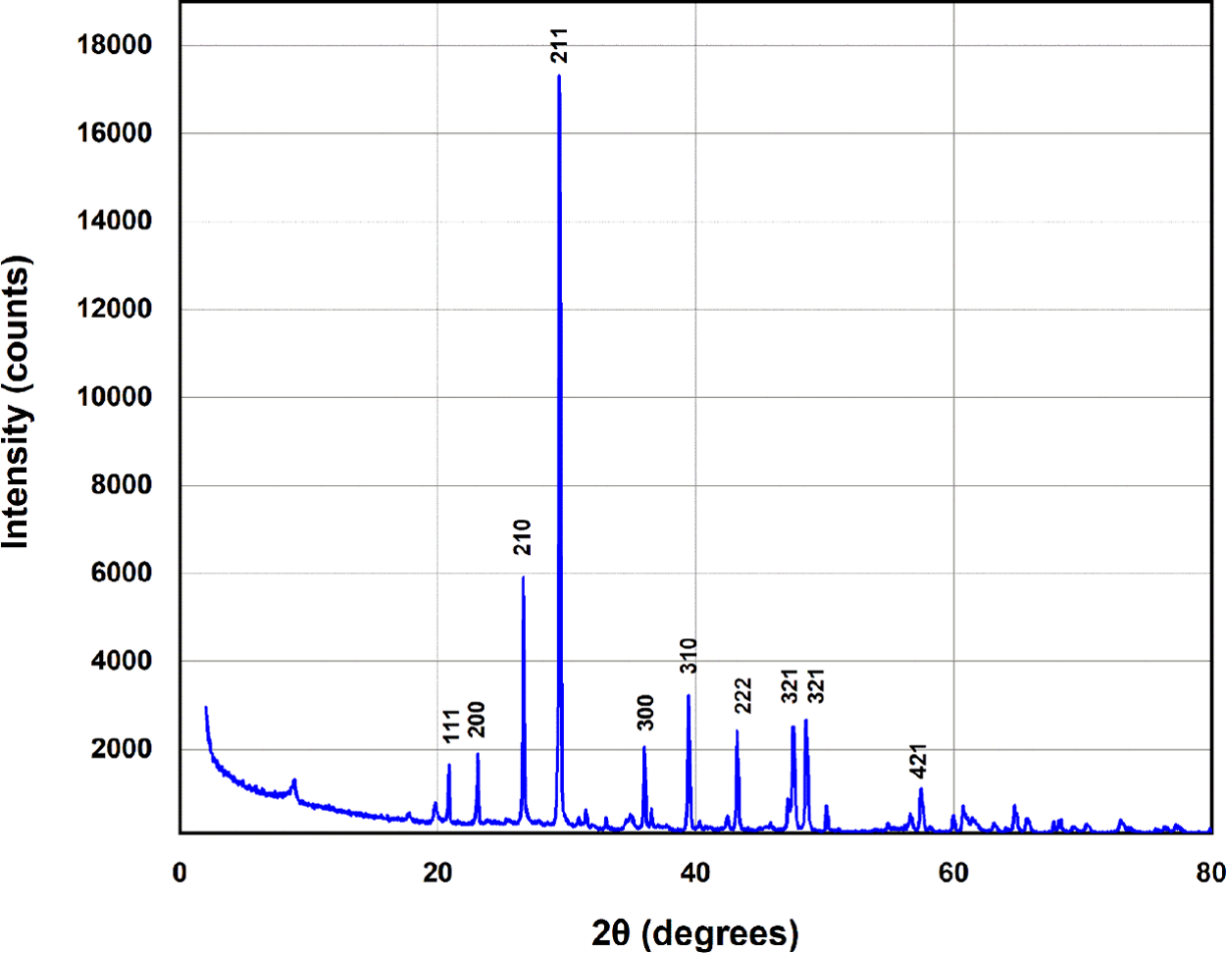
X-Ray diffractometer data of the Marcellus shale powder.

The Malvern Panalytical’s X’Pert^3^ multipurpose X-ray diffraction system was used for mineralogy analyses of the Marcellus shale sample to investigate the mineralogy that could have affected adsorption characteristics. Figure 2 shows the XRD data for the Marcellus shale sample. A computer program (RockJock) was used to give a quantitative estimation for the mineralogy of the sample.

Table 2 illustrates the resulting percentage of the mineral contents. The XRD results show that quartz and illite represent the majority of minerals of the sample tested. Chlorite, kaolinite, and carbonates have also been detected in the sample but in lower quantities. Thus, it is expected that the major contributor to the polymer adsorption is the illite and the montmorillonite, since quartz has a relatively low surface area. Furthermore, the polymer adsorption in the organic content of the shale was neglected as indicated by previous work in which the clay content of shales is the major contributor to the polymer adsorption.^37^

**Table 2:** Quantitative Estimations of the Minerals Detected in the Rock and Crushed Core Using the Standardless Option of the ROCKJOCK Program

type	mineral	weight %
silica	quartz	35.6
carbonate	calcite	3.5
	dolomite	2.8
clay	illite	39.4
	montmorillonite	12.1
	kaolinite	6.6

### 3.3 Morphology

**Figure 3:**
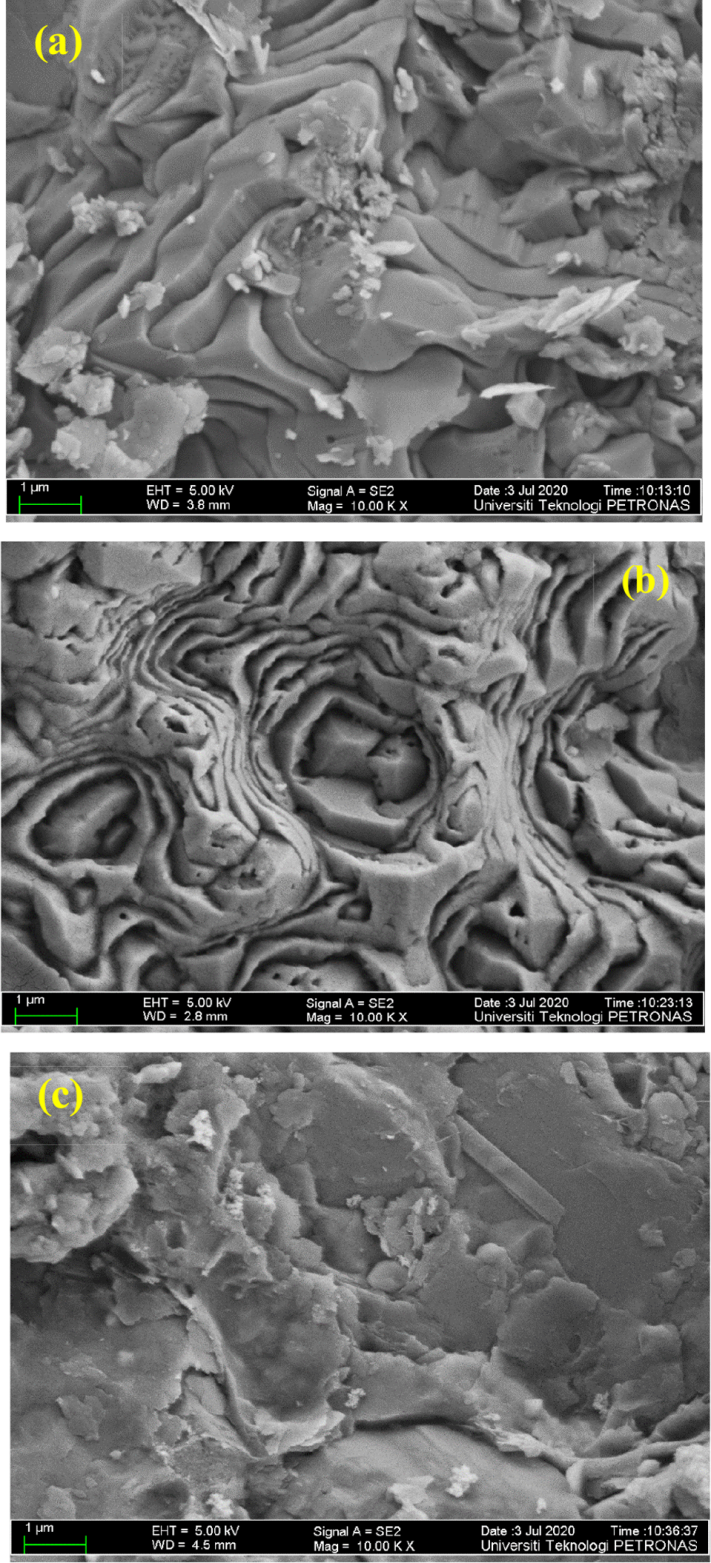
FESEM images of (a) dry Marcellus shale, (b) aged Marcellus shale with the polymer, and (c) aged Marcellus shale with silica nanoparticles.

A field emission scanning electron microscope (FESEM) was used to compare the morphology of the Marcellus shale sample in its dry state after aging with the polymer and after aging with the polymer solution with the nanoparticle. The FESEM image in Figure 3a illustrates the morphology of dry Marcellus shale powder, Figure 3b illustrates an FESEM image after aging the samples with the polymer, and Figure 3c shows an FESEM image after aging the samples with the nanoparticle. Aging of Marcellus shale in polymer solutions resulted in the formation of layers of polymer molecules (adsorbed polymer) (Figure 3b). Similarly, the nanoparticles formed a denser layer, which might be a restriction to the interaction between the polymer molecules and the rock surface as well as occupying some of the adsorption sites, as shown in Figure 3c, which causes a reduction in polymer adsorption on the Marcellus shale sample.

**Figure 4:**
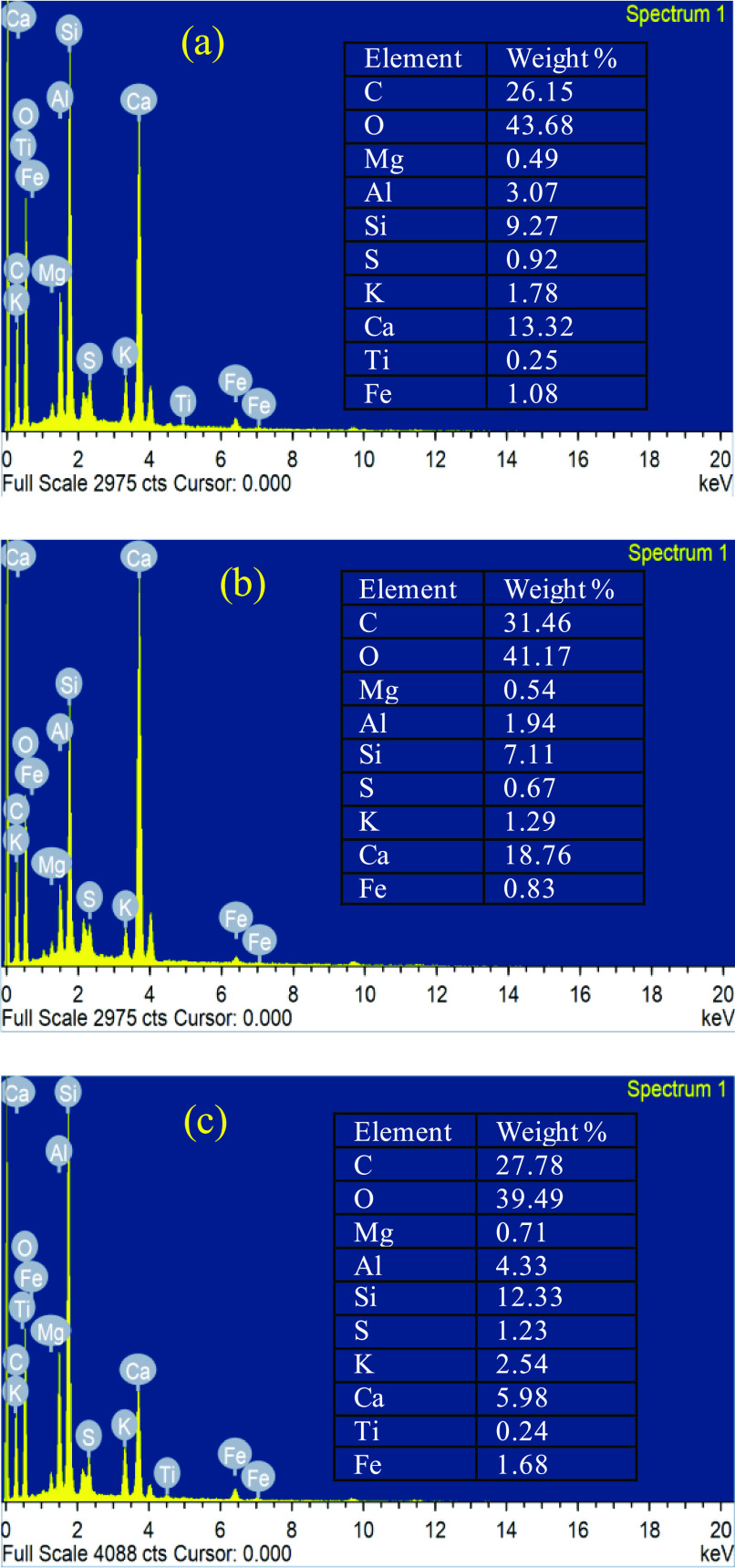
EDX images of (a) dry Marcellus shale, (b) aged Marcellus shale with the polymer, and (c) aged Marcellus shale with the silica nanoparticle.

Energy-dispersive X-ray (EDX) spectroscopy was also used as an extra analytical technique for determining the elements in the three Marcellus samples: dry state, after aging with the MMW polymer, and after aging with the MMW polymer solution with the nanoparticle. Figure 4 shows the EDX data of the three samples, which illustrates the presence of the adsorbed polymer on the Marcellus sample aged with the MMW polymer. This is noted mainly by the increase in the carbon weight %. It is also noted that the silica content in Figure 4c is higher due to the introduction of the silica nanoparticles into the shale surface. This could mean that some of the silica nanoparticles will be retained in the Marcellus shale, which shields the wall of the sample reducing the surface available for the polymer to be adsorbed.

### 3.4 Particle Size and Zeta Potential of the Silica Nanoparticles

Due to the small size of nanoparticles, they can penetrate some of the fractures/pores, which some of the additives in the injected fluids are not able to access. Therefore, nanoparticles can provide a better fracturing efficiency for the smaller fractures because of the following reasons:

- Nanoparticles such as polyelectolyte complexes can be used as gel breakers.
- Nanoparticles can be used to reduce the fracturing fluid filtrate rate to the matrix (leaking-off).
- They can also be used as a nanoproppant preventing collapse of fractures and closure of the nanosized or microsized fissures.
- They can be used as nanocrosslinkers.

**Figure 5:**
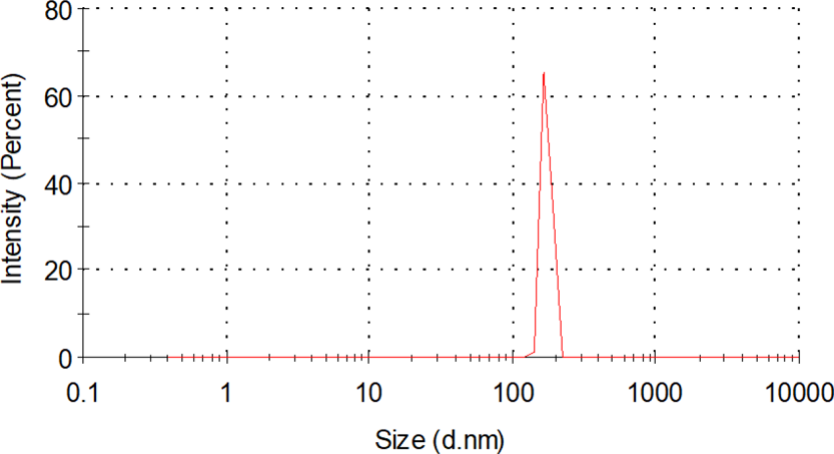
Silica nanoparticle size distribution.

Figure 5 shows the size distribution of the silica nanoparticles used in this study with a diameter size of approximately 160 nm.

Moreover, polymer adsorption on silica nanoparticle surfaces was investigated by observing the zeta potential, using dynamic light scattering. The zeta potential was considered, as the UV–vis instrument was not able to detect the small changes in the polymer concentration before and after interaction with the nanoparticle.^38^ The silica nanoparticles’ zeta potential in solutions was ranging from an average of −35 mV, for the nanoparticles’ solutions before exposure to the polymer, to −62.7 mV, for the nanoparticles’ solution before exposure to the polymer. The results illustrate an adsorption trace of polymer molecules on the surfaces of the silica nanoparticles, as suggested by the zeta potential. However, the polymer adsorption on the silica nanoparticles is neglected in the following adsorption tests, as it is difficult to detect the adsorption quatitatively using the UV–vis.

### 3.5 Polymer Solution Rheology

**Figure 6:**
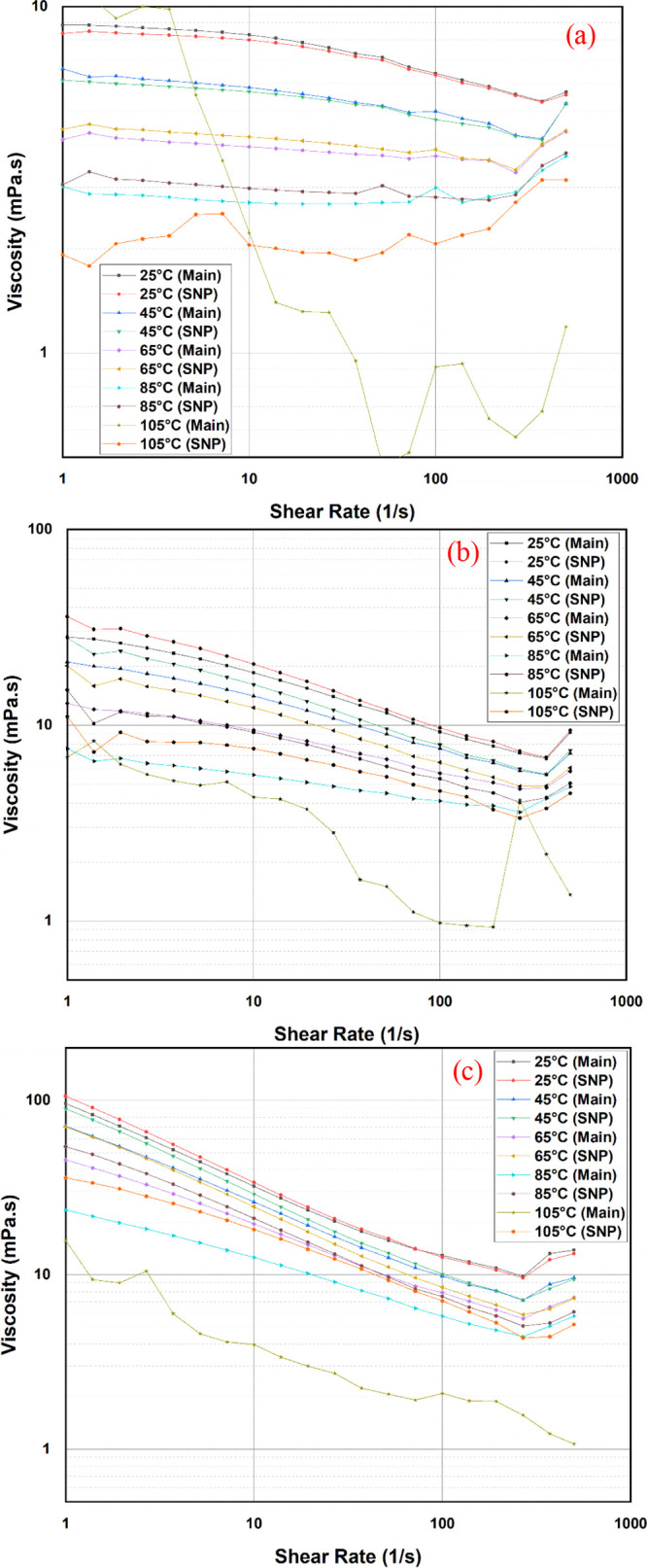
Rheology behavior of (a) LMW, (b) MMW, and (c) HMW polymers at different temperatures with (SNP) and without (Main) silica nanoparticles.

Figure 6 illustrates the rheological study data at concentrations of 1000 and 2500 ppm KCl of the three polymers: Flopaam 3130 S, Flopaam 3330 S, and Flopaam 3630 S. The three polymers’ solution generally exhibited non-Newtonian (shear thinning) behavior in the range of 0.1 and 350 s^–1^ shear rate, after which a slight increase was observed showing a shear thickening behavior. It is generally understood that shear thinning is due to the more significant breakage/destruction of the entanglements between the polymer molecules at a higher shear rate. Consequently, a decrease occurs in the hydrodynamic size of the polymer, and there is thereby a reduction in polymer viscosity.^17,39^ Nevertheless, it is possible that the addition of some nanoparticles can cause the polymer chains to physically adsorb on the nanoparticle surfaces.^40,41^Figure 6 shows a comparison for the polymer rheology with and without silica nanoparticle solution. Overall, it is possible to say that polymers with higher molecular weights are more sensitive to shear, as the viscosity of the higher molecular weight decreased almost 10 times with shear. It is observed that the addition of silica nanoparticles generally resulted in an increase in the viscosity of the polymers. This is explained by the interaction of the polymer chains, causing intercalation of the polymer molecules. This might occur when the polymer molecules stack between the nearest nanoparticles without forming a covalent bond. Another explanation is the molecules’ adsorption on nanoparticle surfaces, leading to a 3D network structure formation, as explained by Fakoya.^41^ The shear thickening behavior results at a higher shear rate, which is possibly due to the nature of some of the existing coiled polymer molecules showing a higher resistance to flow than stretched ones.

Thus, a greater pressure gradient or force will be needed for forcing the coiled molecules to flow with the increase of the shear rate.^42^

### 3.6 The Effect of Temperature

Figure 6 also shows the effect of the temperature (25, 45, 65, 85, and 105 °C) on the rheology of the three polymers. It can be observed that initially, at a lower temperature, the behavior of the polymers with and without the nanoparticles is almost similar, decreasing with the shear rate. The decrease in the viscosity of the three polymers with temperature is because of the gradual degradation with the increase of the temperature. After reaching a temperature of 65 °C and above, the polymer solution tends to lose much of its viscosity until it fails at 105 °C due to the degradation of the polymer. On the other hand, the polymer that was mixed with the nanoparticle exihibited a more stable behavior at elevated temperature (105 °C) for the three polymers. This can also be explained with the same reason mentioned previously, i.e., by the intercalation caused by the polymer molecules and the adsorption of the molecules on nanoparticle surfaces.

### 3.7 Polymer Adsorption Kinetics

**Figure 7:**
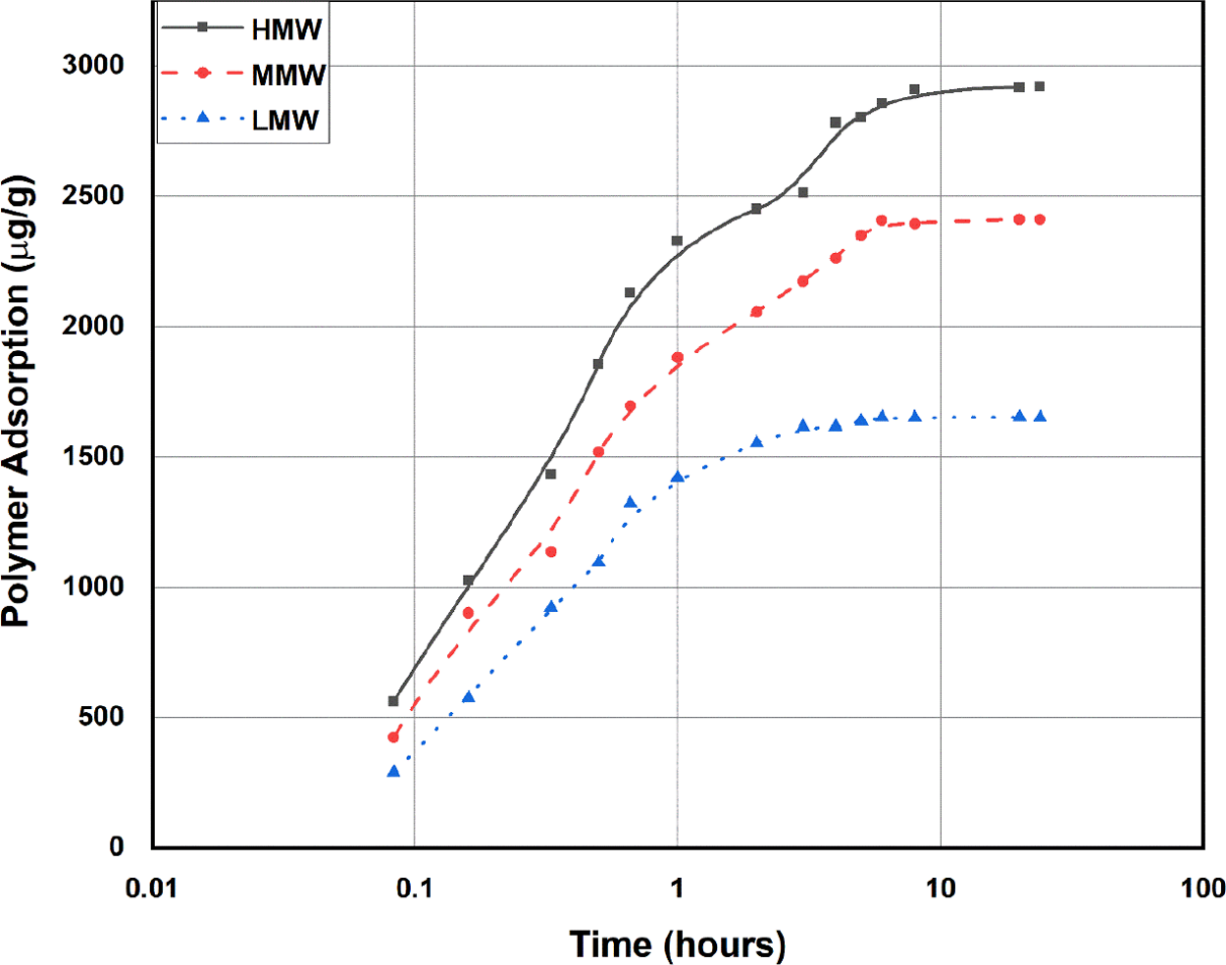
Adsorption kinetics for LMW, MMW, and HMW in Marcellus Shale.

The polymer adsorption kinetics on the Marcellus shale sample were also investigated at a temperature of 65 °C to determine the time required for equilibrium at which most of the adsorption will be achieved. The polymer adsorption at a concentration of 1000 ppm for the three polymers was determined at 14 different time periods (0.083, 0.166, 0.33, 0.5, 0.66, 1, 2, 3, 4, 5, 6, 8, 20, and 24 h) at a solid-to-liquid ratio of 0.1. This was done using static adsorption tests by taking 3 mL of the polymer solution at the predetermined times. Figure 7 shows the trend of the time required for the adsorption to reach the equilibrium. The adsorption started with a steep linear increase in the first 0.5 h, achieving more than 50% of the total adsorption. Then, adsorption kept increasing but less considerably till it started to flatten after almost 4 h for the three polymers. Adsorption data were further observed for the next 20 h, showing a relatively insignificant increase in adsorption. Therefore, 4 h of adsorption time was given for the static tests conducted to ensure that much of the adsorption will be achieved. Figure 7 also shows that the highest adsorption was in the polymer with the highest molecular weight. The main reason for the high adsorption capacity in the HMW polymer might be due to the nature of the polymer molecules. Polymer molecules vary in size with molecular weight. At LMW, the polymer has shorter molecules in length compared to higher molecular weights, which have extended and longer molecules. In fact, the length of the polymer determines its weight. Therefore, even if the number of adsorbed polymer molecules is the same for high and low molecular weights, the adsorption reported in micrograms of the polymer will show a higher polymer adsorption.

### 3.8 Adsorption Tests

Due to the high-pressure requirement for fracturing and the complexity of fracture distributions and directions in the dynamic studies, we used the static adsorption tests to investigate and compare the polymer adsorption on the Marcellus shale using eq 1.

#### 3.8.1 The Effect of Polymer Concentration

##### 3.8.1.1 Polymer Adsorption at Different Polymer Concentrations

**Figure 8:**
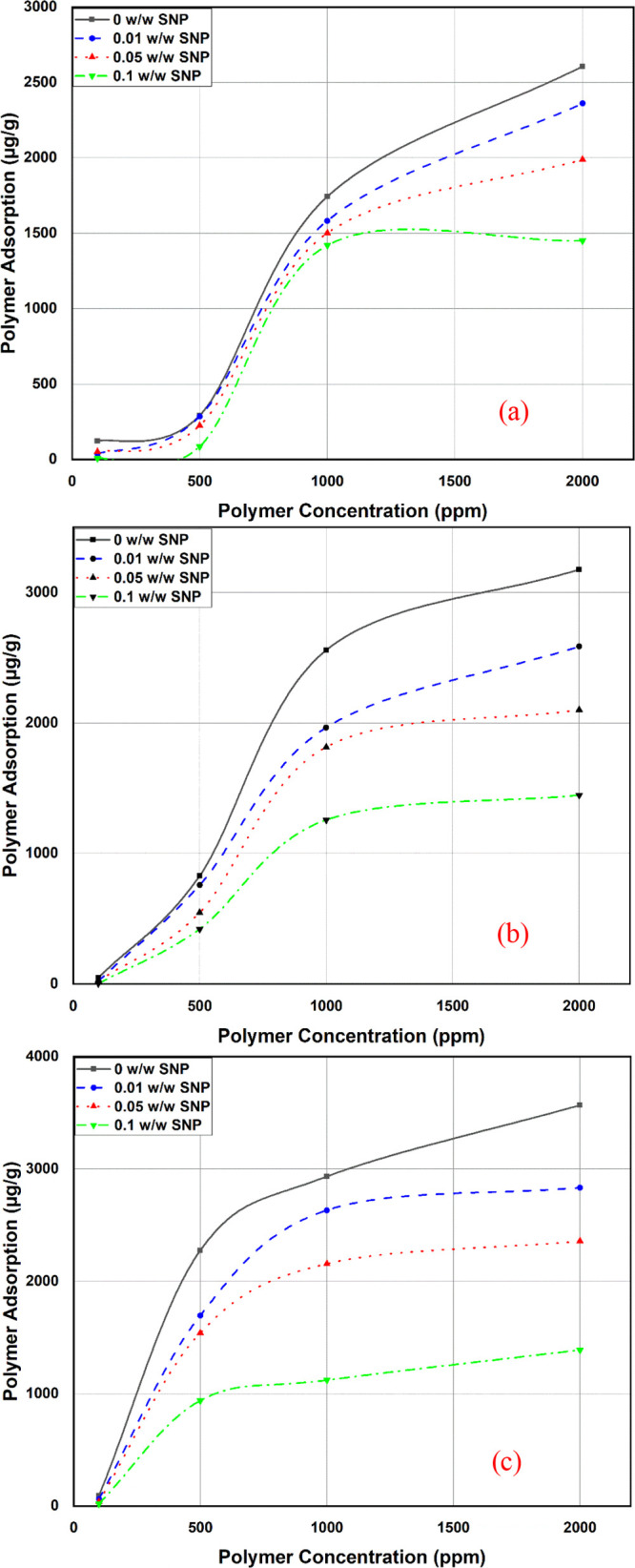
Adsorption reduction at various polymer concentrations: (a) LMW, (b) MMW, and (c) HMW.

The effect of nanoparticle concentration on polymer adsorption behavior on the Marcellus shale samples was investigated at a reservoir temperature of 65 °C (Figure 8). Generally, polymer adsorption increases with polymer concentration because of the increase in the numbers of the molecules of the polymer per unit volume with the increasing polymer concentration. This will increase the probability of the adsorption of the polymer on the shale surfaces.

##### 3.8.1.2 Polymer Adsorption Reduction with Nanoparticles

**Figure 9:**
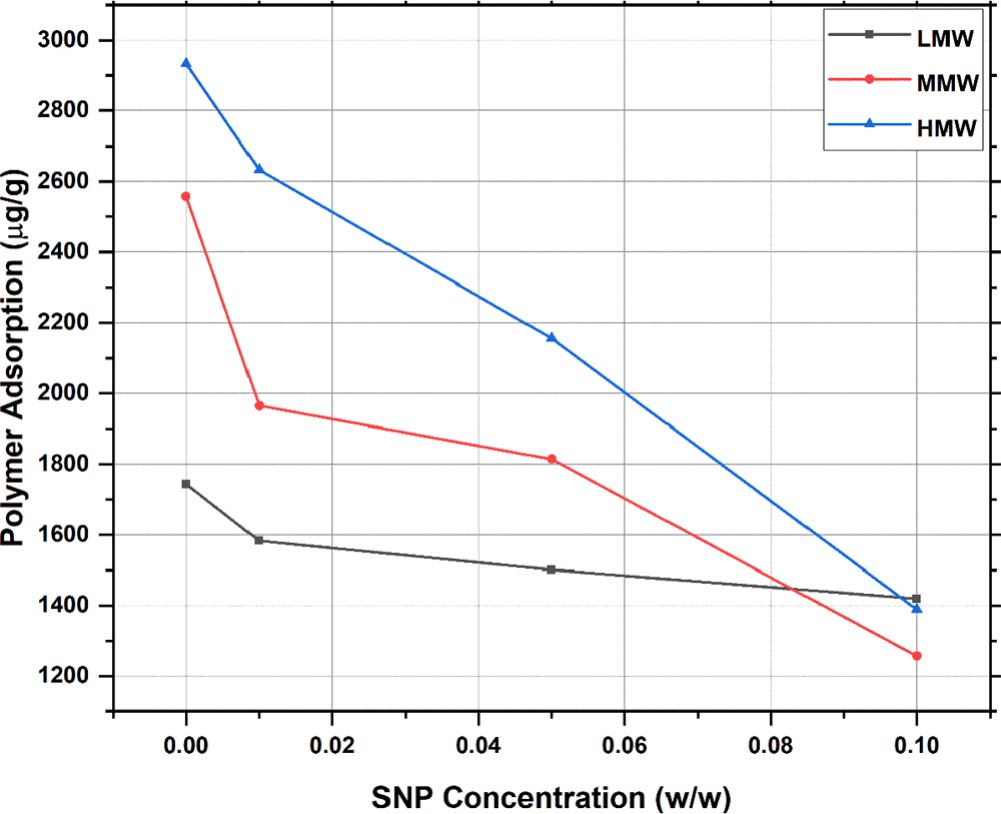
Effect of silica nanoparticle concentrations on the reduction of the three polymers at 2000 ppm polymer concentration and 2500 ppm KCl.

Figure 9 shows how the three polymers’ adsorptions were affected by the addition of 0.01, 0.05, and 0.1 w/w% of the silica nanoparticle to the polymer solutions at polymer concentrations ranging between 100 ppm and 2000 ppm. Adding the nanoparticles has generally significantly reduced the polymer adsorption on the Marcellus shale sample. From Figure 9, the polymer adsorption reduction was observed for the three polymers with an average decrease of almost 54%. It can also be noted that the addition of 0.01 w/w nanoparticles has considerably decreased the adsorption for all three polymers. A further increase in the nanoparticle concentration showed the highest decrease in polymer adsorption, as shown in Figure 9. Figure 9 also shows that the highest polymer adsorption reduction was noted for the HMW, whereas the lowest was in the LMW polymer because less-adsorbed polymers for the HMW will result in higher reduction in the weight of reported adsorption as adsorption is reported in the mass of polymers per mass of rock. This could be the result of the fact that higher molecular weights have higher mass. Thus, when adsorption is reported in the mass of the polymer per mass of rock, it yields relatively higher adsorption.

From the FESEM results, it can be concluded that some of the silica nanoparticles will be retained in the Marcellus shale, which shields the wall of the sample, reducing the surface available for the polymer to be adsorbed. Taking the economic cost into consideration, the highest concentration of the silica nanoparticles was considered to be 0.1.

#### 3.8.2 The Effect of Salinity

##### 3.8.2.1 Polymer Adsorption at Different Salinities

To further investigate the effect of silica nanoparticles on the polymer adsorption, polymer adsorption tests on the Marcellus shale were conducted at 1000 ppm for the three polymers with salinities of 2500, 10,000, 30,000, and 50,000 ppm.

**Figure 10:**
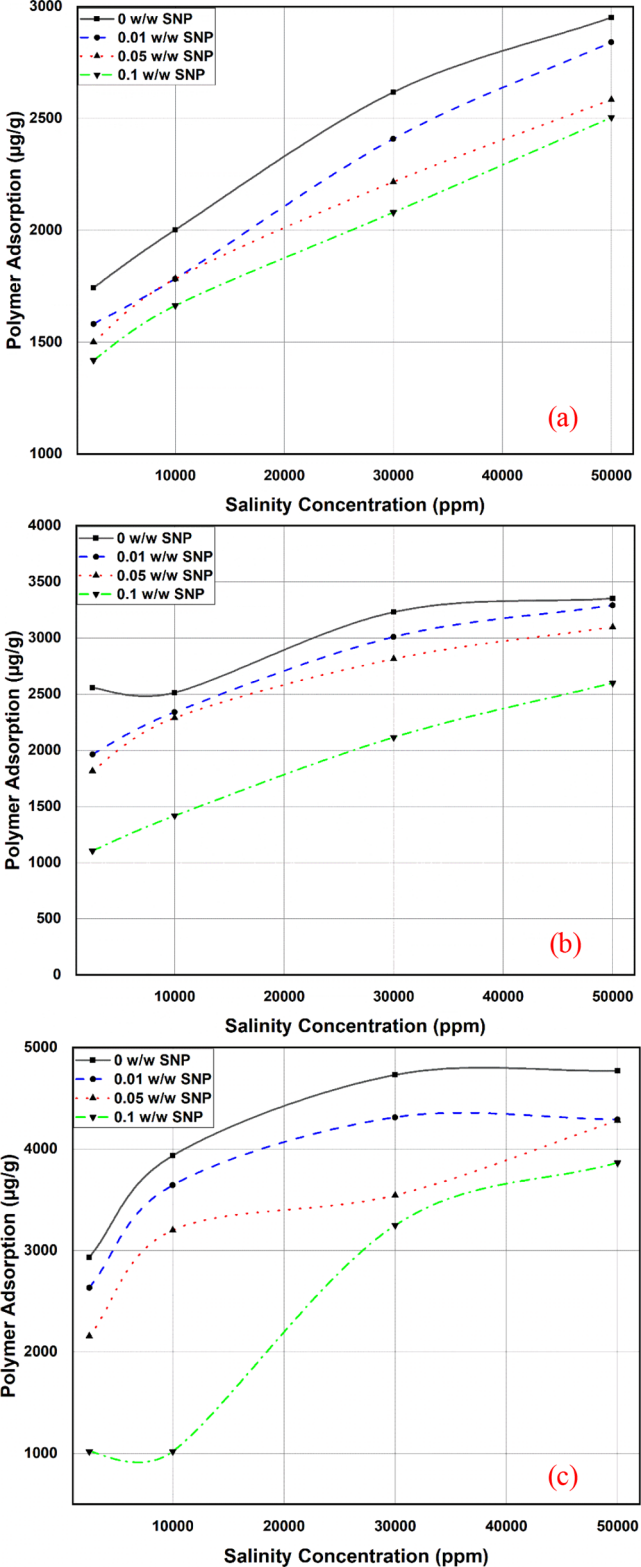
Polymer adsorption and reduction at various KCl concentrations at 1000 ppm of (a) LMW, (b) MMW, and (c) HMW polymer concentration.

Figure 10 shows the effect of salinity on polymer adsorption. The overall trend is an increment of polymer adsorption with salinity and a decrease in adsorption with a nanoparticle concentration increase. The initial increase of polymer adsorption with salinity is reasonably explained by the flexibility of the polymer molecules, which results in higher response to the ionic strength, thereby shrinking the polymer molecule allowing for more volume available for adsorption at the shale surfaces.^43^

##### 3.8.2.2 Polymer Adsorption Reduction with Nanoparticles under Salinity Effect

The decrease in polymer adsorption with silica nanoparticles can also be explained from the FESEM results (Figure 3) in which the silica nanoparticle provides a layer on the solid surface, reducing the adsorption sites available for the polymer and hence preventing more of the polymer molecules to be further adsorbed.

The slight reduction in adsorption despite the increase in salinity, with an increase of the nanoparticle concentration from 0.01 to 0.1 w/w to almost 20% reduction, could be explained by the nanoparticles’ repulsive forces diffusing away from the bulk solution to the clay’s surfaces.^44^ This is because the ion charge’s efficiency to shield the nanoparticles and cause a reduction in the repulsive forces between the nanoparticles will be reduced with increasing the concentration of the nanoparticle. Table 3 lists the highest and lowest polymer adsorptions for each of the three polymers at 1000 ppm polymer concentration.

**Table 3:** Highest and Lowest Polymer Adsorption and Reduction for the Three 1000 ppm Polymers at 50,000 ppm KCl

polymer type	salinity (ppm)	SNP (w/w)	polymer adsorption (μg/g)
Flopaam 3130	50,000	0	2951
	50,000	0.1	2504
Flopaam 3330	50,000	0	3352
	50,000	0.1	2607
Flopaam 3630	50,000	0	4772
	50,000	0.1	3865

It can be observed from Table 3 that, similar to the increase in polymer concentration, the adsorption of the polymer generally increases with molecular weight. Nevertheless, the polymer adsorption reduction was less significant compared to that of the polymer concentration effect. The three polymers showed a polymer adsorption reduction averaged with almost 20% compared to the previous reduction with only polymer concentration effect, which showed a nearly 50% reduction. One possible explanation for this is that although the nanoparticle provided a coating layer on the surface of the Marcellus sample, some of the squeezed polymer molecules are still able to come in contact with the solid surface.

**Figure 12:**
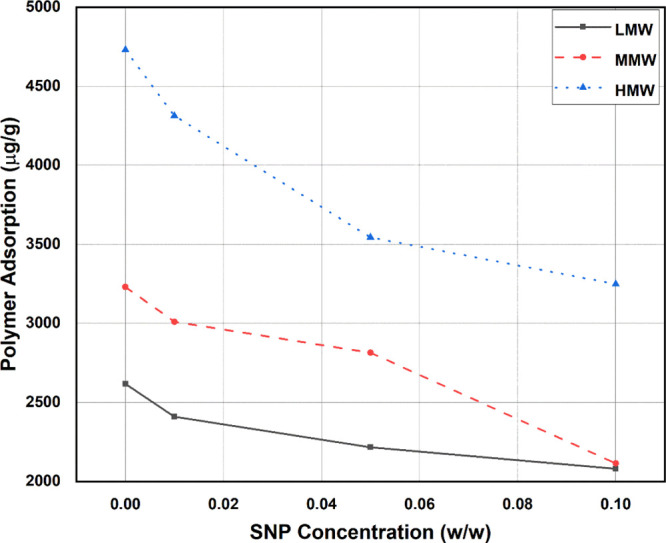
Effect of silica nanoparticle concentrations on the reduction of the three polymers at 1000 ppm polymer concentration and different KCl concentrations.

Figure 12 shows the trend of polymer adsorption reduction at KCl 30,000 ppm with an increase of the silica nanoparticle concentration. This point was selected because the adsorption and adsorption reduction peaked at 30,000 ppm KCl, after which increasing salinity did not show significant changes in the adsorption and adsorption reduction, especially for the MMW and HMW polymers, as illustrated in Figure 10.

### 3.9 Results and Summary

The ability of silica nanoparticles to improve polymer rheology and adsorption reduction in Marcellus shale samples at reservoir temperature was investigated. Three polymers of low, medium, and high molecular weights (1–12 MD) were used in this study. A polymer concentration of 100–2000 ppm and salinity of 2500–50,000 ppm were used to investigate the amount of the adsorption. Furthermore, silica nanoparticles of 0.01–0.1 w/w were used to investigate the polymer rheology improvement and the polymer adsorption reduction. Overall, the three polymers (LMW, MMW, and HMW) are sensitive to shear with a significant decrease in their viscosity. However, the addition of silica nanoparticles generally resulted in a less shear effect on the polymer. Moreover, the addition of the nanoparticle showed a better temperature tolerance of the three polymers at as high as 105 °C. From the kinetic study, it is observed that 4 h of adsorption time was enough for the three polymers to reach adsorption equilibrium. Polymer adsorption was shown to increase with the polymer concentration, but adding a certain amount of nanoparticles results in the reduction in the polymer adsorption on the Marcellus shale. Furthermore, high polymer adsorption was observed with increasing salinity. However, adsorption decreased with the addition of the nanoparticles. Polymer adsorption reduction reached as high as 50% of the polymer adsorption with the use of nanoparticles in the polymer solution. This shows how the addition of nanoparticles to the fracturing fluid can be significant in terms of improving its efficiency. Further investigation on the polymer adsorption reduction using nanoparticles at high pressure and different flowrates is recommended for future study.

## 4 Conclusions

In this study, static adsorption of a low, medium, and high molecular weight HPAM with and without silica nanoparticles was investigated on the Marcellus shale sample at a reservoir temperature of 65 °C. The rheological behavior of the three polymers investigated was also compared before and after the addition of the silica nanoparticles at 25, 45, 65, 85, and 105 °C. The advantages of using nanopartilces in polymeric fracturing fluids are twofold, improving the rheologial properties of the polymer and reducing the polymer adsorption. The following observations were made:

- The addition of silica nanoparticles resulted in polymer adsorption reduction on the Marcellus shale sample to almost half. This can be related to the reduced surface area on the Marcellus shale surface occupied by the silica nanoparticles. Thus, the probability of polymer and shale contact was considerably reduced.
- Polymer adsorption and adsorption reduction were significantly affected by the increased polymer concentration.
- The highest polymer adsorption was observed for the highest molecular weight, whereas the lowest was observed for the lower molecular weight polymer.
- The addition of silica nanoparticles to the polymer solution improves the polymer rheology, particularly at elevated temperatures.
